# A qualitative exploration of contextual factors that influence dissemination and implementation of evidence-based chronic disease prevention across four countries

**DOI:** 10.1186/s12913-018-3054-5

**Published:** 2018-04-02

**Authors:** Elizabeth L. Budd, Anna J. deRuyter, Zhaoxin Wang, Pauline Sung-Chan, Xiangji Ying, Karishma S. Furtado, Tahna Pettman, Rebecca Armstrong, Rodrigo S. Reis, Jianwei Shi, Tabitha Mui, Tahnee Saunders, Leonardo Becker, Ross C. Brownson

**Affiliations:** 1 Prevention Research Center in St. Louis, Brown School, Washington University in St. Louis, One Brookings Dr., Campus Box 1196, St. Louis, MO 63130 USA; 2 Shanghai Tenth People’s Hospital, Tongji University School of Medicine, No .1239 Siping Road, Yangpu District, Shanghai, China; 3 The Hong Kong Polytechnic University, GH 410 Department of Applied Social Sciences, Hung Hom Kowloon, China; 4 Melbourne School of Population and Global Health, The University of Melbourne, Level 5, 207 Bouverie Street, Victoria, 3010 Australia; 5 Pontifical Catholic University of Parana, Curitiba, Rua Imaculada Conceicao, 1155, Prado Velho, 80215901 Brazil; 6 Department of Surgery (Division of Public Health Sciences) and Alvin J. Siteman Cancer Center, Washington University School of Medicine, Washington University in St. Louis, St. Louis, MO 63130 USA; 7 Prevention Science Institute, College of Education, University of Oregon, 5261 University of Oregon, Eugene, OR 97403 USA

**Keywords:** Australia, Brazil, China, United States, Chronic disease, Evidence-based, Implementation, Dissemination

## Abstract

**Background:**

Little is known about the contextual factors affecting the uptake of evidence-based chronic disease interventions in the United States and in other countries. This study sought to better understand the contextual similarities and differences influencing the dissemination and implementation of evidence-based chronic disease prevention (EBCDP) in Australia, Brazil, China, and the United States.

**Methods:**

Between February and July 2015, investigators in each country conducted qualitative, semi-structured interviews (total *N* = 50) with chronic disease prevention practitioners, using interview guides that covered multiple domains (e.g., use of and access to EBCDP interventions, barriers and facilitators to the implementation of EBCDP interventions).

**Results:**

Practitioners across the four countries reported only a few programmatic areas in which repositories of EBCDP interventions were used within their workplace. Across countries, academic journals were the most frequently cited channels for accessing EBCDP interventions, though peers were commonly cited as the most useful. Lack of time and heavy workload were salient personal barriers among practitioners in Australia and the United States, while lack of expertise in developing and implementing EBCDP interventions was more pertinent among practitioners from Brazil and China. Practitioners in all four countries described an organizational culture that was unsupportive of EBCDP. Practitioners in Brazil, China and the United States cited an inadequate number of staff support to implement EBCDP interventions. A few practitioners in Australia and China cited lack of access to evidence. Partnerships were emphasized as key facilitators to implementing EBCDP interventions across all countries.

**Conclusions:**

This study is novel in its cross-country qualitative exploration of multilevel constructs of EBCDP dissemination and implementation. The interviews produced rich findings about many contextual similarities and differences with EBCDP that can inform both cross-country and country-specific research and practice to address barriers and improve EBCDP implementation among the four countries long-term.

## Background

The majority of premature deaths in middle- and high-income countries are due to cancer and other chronic diseases such as type 2 diabetes and heart disease [1]. An emerging evidence-base has occurred in response to this global increase in chronic diseases. The practice of evidence-based chronic disease prevention (EBCDP) integrates science-based interventions and community preferences in order to improve population health, and when it is applied, can prevent many cases of morbidity and mortality due to chronic disease [2]. In order to practice EBCDP, public health practitioners must use evidence to inform decisions about how to improve their performance as health professionals and population health outcomes [3].

Well-recognized reviews document dozens of interventions known to be effective at preventing cancer and other chronic diseases in middle- and high-income countries, yet these interventions are not widely used [4–10]. Studies have identified a wide variety of contextual factors that influence the use of EBCDP [11–17]. Research primarily from the United States, Australia, and Canada has focused on personal and organizational-level barriers and facilitators to EBCDP. Examples of barriers to EBCDP include: lack of time, lack of skills and formal training related to the evidence-based decision-making process, lack of networks for support, lack of incentives to use evidence in decision-making, lack of funding, and an unsupportive organizational culture around the use of evidence [18–23]. Examples of facilitators to EBCDP include: easy access to evidence, training of staff and organizational leaders on the evidence-based decision-making process, opportunities to network with peers, and collaboration across research and practice sectors [18–23]. Despite a global need for EBCDP, and several studies calling for an action plan to better address the burden of chronic disease around the world [24–26], little is currently known about how access to EBCDP interventions, as well as personal and organizational-level barriers and facilitators of EBCDP vary by middle- and high-income countries and how these differences might affect the transfer and translation of evidence-based interventions within and across countries [24, 26–28]. The objective of this qualitative study is to explore the channels chronic disease practitioners in Australia, Brazil, China, and the United States use to access EBCDP in addition to, personal and organizational-level barriers and facilitators that they perceive influence the dissemination and implementation (D&I) of EBCDP.

Australia, Brazil, China, and the United States were selected for this study based on their positions as opinion leaders in their regions [29–33], their variation on important contextual variables (e.g., organizational management practices, policy factors), and the high rates of chronic diseases prevalent in each country [11]. In 2014, 91% of deaths in Australia, followed by 88% in the United States, 87% in China, and 74% in Brazil were attributed to chronic diseases [34]. Middle-income countries, Brazil and China, were chosen due to the sparse literature [35, 36] and D&I of EBCDP there compared to higher-income countries [6, 12, 37–40]. There is currently limited knowledge of the variation in contextual variables and D&I within such countries [11, 16, 28, 41, 42] as well as understanding of the approaches that might be translated across countries (e.g., adaptation and scaling up) in order to affect population prevalence rates of chronic diseases [16, 17, 24]. Exploring differences in contextual variables across the four countries also contributes to building critical knowledge that should set the stage for future measurement development and projects allowing scale-up of findings.

## Methods

### Data collection

A total of 14 investigators from Australia, Brazil, China, and the United States working in academic settings developed a semi-structured interview guide, informed by a narrative review of existing EBCDP instruments and gaps in the literature [18–20, 22, 43–52]. The interview guide included six major domains: 1) biographical information and experience (e.g., age, gender, degree); 2) awareness of the existence of EBCDP interventions (e.g., To what extent have evidence-based interventions been used to support or enhance decision-making in your organization over the past 12 months?); 3) barriers to implementing EBCDP interventions (e.g., Overall, can you identify any personal barriers that impede your ability as an individual to implement EBIs or make evidence-based decisions?); 4) policy climate and support (e.g., Think about the evidence-based intervention you described earlier. Can you think of things that helped you to implement these interventions?); 5) administrative support (e.g., How would you describe the culture/climate of your department as it relates to implementing evidence-based processes?); and 6) D&I strategies (e.g., What avenues allow you to learn about the current findings in evidence-based interventions?). To minimize the risk of bias deriving from the research team, the interview guide was reviewed by an expert panel of seven consultants who work in chronic disease prevention, and it underwent pilot testing to gather feedback in each country. Pilot testing also included forward and backward translation for content and meaning in China and Brazil. Revisions were made to the interview guide based on feedback to improve clarity of questions.

Purposive sampling was used to select respondents. In each country, we identified comparable respondents based on which organizations had primary authority for chronic disease prevention and control, and where most funding for EBCDP was applied, thereby making our cross-country results as analogous as possible. These organizations were local health departments in the United States; community health centers, hospitals, and the Centers for Disease Control and Prevention in China; regional state-based health departments and community health services in Australia; and local health departments and the Ministry of Health in Brazil. From each organization, a list of eligible participants was enumerated. A maximum of two attempts were made to reach these key informants before an attempt was made to reach an alternate respondent. Additional eligibility criteria included: ages 21 years and older, at least 6 months working for their organization of employment, and ability to speak one of three languages (English, Putonghua, or Portuguese).

Ethics approval was granted by the universities of the investigators involved in data collection including, The University of Melbourne Human Ethics Committee, Pontifica Universidade Catolica do Parana Research Ethics Committee, The Hong Kong Polytechnic University Human Ethics Committee of the Faculty of Health and Social Science, and Washington University in St. Louis Institutional Review Board. Trained research team members conducted the interviews via telephone with the exception of the interviews in China, which were conducted in-person as a culturally tailored interview strategy. Interviews were conducted in the native language, audio recorded, transcribed, and translated to English when necessary. All participants were asked to review a written consent information sheet upon recruitment to the study. Verbal consent was provided by all participants. Documentation of verbal consent was waived by the aforementioned ethics committees. A goal of 12 interviews per country (total *N* = 48) was anticipated based on previous research showing that when the subject being investigated is relatively narrow in focus and the interview (sub)sample is relatively homogenous, meaningful themes can be developed after six interviews and saturation may be present with as few as 12 interviews [53, 54]. Interviews were carried out and analyzed continuously, with more interviews being conducted until thematic saturation had been reached. All interviews took place between February and July of 2015.

### Analysis

In this framework analysis [55], three trained researchers (the project coordinator and two graduate research assistants) in the United States reviewed the data with the exploratory research aims in mind and using the interview guide domains as an a priori organizational framework. That is, there was a hierarchical coding structure: all themes deductively identified by the researchers were coded in the codebook as child/sub codes of their respective a priori interview guide domain parent codes, when relevant. The codebook with thematic code definitions and examples were created using NVivo 10 software. NVivo was also used to analyze the data. The first transcript from each country was triple coded (i.e., coded independently by the three researchers) and the results were compared and discussed until a consensus was reached. The researchers continued to triple code the interviews from each country until 90% inter-rater agreement was demonstrated (*N* = 2–5 transcripts/country). Once consistency in coding was reached each transcript was coded by one of the three researchers. Thematic development and coding occurred concurrently with ongoing interviews. The lack of new coded themes after two transcripts in a row was one signal to the researchers that the interviews had reached saturation on the research topic and no further interviews were conducted. Feedback on the codebook and results was gathered from all of the investigators across the four countries to reduce cross-cultural misinterpretations. Coded themes were then analyzed for patterns, consistencies within and across countries, importance, and if they were new to the literature.

## Results

With 13 interviews in Australia, nine in Brazil, 16 in China, and 12 in the United States (total *N* = 50; mean duration = 27 min) thematic saturation was reached. Most of the public health practitioners interviewed were between 30 and 49 years old (66%) and female (84%), though practitioners in Brazil were younger (56% between 30 and 39 years) and evenly split by gender. The majority of the practitioners held graduate degrees, most commonly in public health. Participating practitioners from Brazil tended to have more education than those from the other countries. For instance, four of the nine practitioners from Brazil held a PhD, compared with only one practitioner from Australia, one from the United States, and none from China. Most practitioners from Australia, Brazil, and the United States worked as public health educators, managers, or program coordinators, whereas the largest contingents of practitioners from China were physicians or refused to disclose their employment title. Table 1 outlines biographical information of respondents from each country. A summary of the results appears in Table 2.

**Table 1:** Biographical Information on the Study Sample of Practitioners Working in Chronic Disease Prevention (*N* = 50)

	Australia *N* = 13	Brazil *N* = 9	China *N* = 16	United States *N* = 12
Gender				
Female	92% (12)	55% (5)	81% (13)	100% (12)
Male	8% (1)	45% (4)	19% (3)	0% (0)
Age				
21–29	8% (1)	0% (0)	0% (0)	8% (1)
30–39	23% (3)	56% (5)	44% (4)	33% (4)
40–49	23% (3)	22% (2)	25% (4)	42% (5)
50+	31% (4)	22% (2)	25% (4)	16% (2)
Refused	15% (2)	0% (0)	6% (1)	0%(0)
Education				
High School or Less	0% (0)	0% (0)	6% (1)	0% (0)
Some college	0% (0)	0% (0)	6% (1)	0% (0)
College degree	23% (3)	33% (3)	25% (5)	8% (1)
Graduate degree	69% (9)	67% (6)	30% (6)	92% (11)
Missing	8% (1)	0% (0)	12% (2)	0% (0)
Employment title				
Clinical Management	0% (0)	0% (0)	12% (2)	0% (0)
Community health nurse	0% (0)	11% (1)	0% (0)	0% (0)
Department head	0% (0)	11% (1)	12% (2)	16% (2)
Physician	0% (0)	0% (0)	30% (6)	0% (0)
Program Manager/ Coordinator/Health Educator	64% (8)	67% (6)	12% (2)	72% (9)
Statistician	0% (0)	11% (1)	0% (0)	0% (0)
Other	23% (3)	0% (0)	0% (0)	8% (1)
Missing	15% (2)	0% (0)	24% (4)	0% (0)

**Table 2:** Summary of similarities and differences of contextual factors identified across countries

	Australia *N* = 13	Brazil *N* = 9	China *N* = 16	United States *N* = 12
The most commonly cited channels for obtaining information on EBCDP interventions				
Academic journals	x		x	
Conferences	x		x	
Networks	x	x		x
Professional associations	x	x		x
The most *useful* channel for accessing EBCDP interventions was their peers	x	x	x	x
Reported only a few programmatic areas in which evidence-based repositories were being used within their organizations of employment	x	x	x	x
Perceived personal-level barriers to the implementation of EBCDP interventions				
Lack of time	x		x	x
Heavy workload	x			x
Lack of expertise with developing and implementing EBCDP interventions		x	x	
Optimism and versatility in overcoming barriers		x		
Perceived organizational-level barriers to the implementation of EBCDP interventions				
Unsupportive workplace cultures	x	x	x	x
Perceived lack of support for EBCDP from the organization’s leadership			x	
Lack of communication across various groups			x	x
Lack of a workplace policy, mechanism, or incentive to promote and/or keep staff members accountable for making evidence-based decisions in their work	x		x	
Presence of workplace policies that limit personal authority to select the best interventions or to make other changes necessary to incorporate EBCDP	x	x		
Inadequate number of staff to implement EBCDP interventions		x	x	x
Lack of access to evidence	x		x	
Lack of evidence relevant to rural communities				x
Facilitators to implementing evidence-based interventions				x
Funding agencies that require EBCDP interventions				
Having an education/degree	x	x		x
Partnerships/support from others	x	x	x	x

*EBCDP* evidence-based chronic disease prevention; x indicates a salient theme among practitioners in that country

### Channels for learning about evidence-based interventions

Academic journals and conferences were the most commonly cited channels for obtaining information on EBCDP interventions among practitioners in Australia, China, and the United States. Professional networks and associations were also commonly cited among practitioners from Australia, Brazil, and the United States. Overall, practitioners from all four countries reported only a few programmatic areas in which evidence-based repositories (i.e., databases with evidence-based interventions and policies [5, 10]) were being used within their organizations of employment. Practitioners from all four countries agreed that the most *useful* channel for accessing EBCDP interventions was their peers.

*“Networks are most useful because they are a way to hear about research that's been conducted long before it's reported in the peer reviewed literature.” [Australia]*

*“Communication between peers, which basically takes place through trainings and academic conferences.” [China]*

### Personal barriers to implementing evidence-based interventions

Perceived personal-level barriers were defined as deterrents or conditions that impede the implementation of EBCDP interventions that are specific to the individual [20]. Practitioners in Australia and the United States consistently cited lack of time and heavy workload as personal barriers. Lack of time to keep up with the latest scientific evidence was also cited by practitioners from China.

*“It’s just that we have too much work to do. Doctors and other staff in the community-based health centers are overwhelmed. So, we don’t have extra time to get to learn about new evidence or knowledge. What we read, at most, are those articles related to our routine work.” [China]*

A salient personal barrier reported by practitioners from Brazil and China pertained to lack of expertise with developing and implementing EBCDP. However, practitioners from Australia and the United States, where evidence-based public health practice is more established, did not report struggling with the same lack of expertise.

*“…the lack of ability to develop strategies based on evidence.” [Brazil]*

*“[Personal barriers include] lack of skills to effectively communicate evidence-based strategies to policy makers, lack of skills to effectively develop evidence-based chronic disease programs, and let me see, and lack of decision-making authority to select evidence-based chronic disease programs.” [China]*

While lack of expertise emerged as a barrier, practitioners from Brazil tended to be more optimistic and versatile in solving this barrier than their counterparts from other countries citing, on the whole, fewer personal barriers. When asked to describe any personal barriers, several practitioners from Brazil instead described their practices in overcoming barriers.

*“That’s why I think there is not a barrier, right? I think everything is possible…I think the main thing is you’re always studying, seeking knowledge, exchanging experiences with someone who has implemented effective practices. That worked, succeeded. You will have no problem in developing this type of process.” [Brazil]*

*“I do not see obstacles there…any questions I do not know or skills I do not have, at this point I do not have the answer, but I will seek the answer, either by phone or by email, or the next meeting. One is not without answers.” [Brazil]*

### Organizational barriers to implementing evidence-based interventions

Three types of organizational barriers surfaced that impede the implementation of EBCDP interventions: 1) characteristics of the leadership or organization as a whole; 2) organizational policies; or 3) the lack of organizational resources. The majority of themes within the domain of organizational barriers were consistent across countries.

Leadership and Organizational Culture.

Practitioners in all four countries described a workplace culture that was unsupportive of EBCDP. Unsupportive workplace cultures were characterized as resistant to change, new ideas, new policies, and creative thinking.

*“There might sometimes be organizational cultures that are not so strongly evidence driven.” [Australia]*

*“Yes, especially the resistance of the workers themselves who do not want to change their working processes.” [Brazil]*

*“I would say that they (administrators) are supportive, but you still have to set it within your work, so it’s not to the point where that is the… evidence-based programing would not be the driving force, it would be getting your other work done, that it’s funded, and then if you have time, you can do these other types (evidence-based) of program.” [United States]*

Perceived lack of support for EBCDP from the organization’s leadership was another facet of an unsupportive workplace culture, indicated by practitioners from China.

*“Medical staff actually care about what kind of interventions work and what don’t work. But the administrators care more about getting the work done and achieving their goals. If they can’t get what they want from a certain intervention, they won’t be interested. The leaders have their term in office and want to get things done.” [China]*

Lack of communication across various groups was also a theme that arose among practitioners in China and the United States. In China, practitioners emphasized the lack of partnership and sharing of medical records between general hospitals, which provide more tertiary care, and community hospitals, which provide more primary and secondary care. In the United States, the lack of communication between practitioners and policy makers was identified as problematic to implementation of EBCDP interventions.

*“And under the current national health insurance policy, we [in mainland China] lack a chronic disease management system that’s similar to the diabetes management system in Taiwan. To set up such a system, we first need to establish something like an effective information exchange platform or should we say, become more information-based. At present, inadequate informatization [sharing of patient information] is the biggest obstacle.” [China]*

*“You know… something there…something that we are trying to work on a lot, we do this well, and we know we need to improve is working with partners on those policy change efforts and some of the other things that we cannot necessarily do ourselves. A lot of things are not things that local public health can do, I cannot, you know, raise alcohol taxes or…you know there are a lot of things I can do, but I can work with partners to do that and to promote them.” [United States]*

#### Organizational policies

Practitioners in Australia and China described the lack of a policy, mechanism, or incentive to promote and/or keep staff members accountable for making evidence-based decisions in their work as a barrier.

*“The lack of incentive or reward for using evidence-based decision making is definitely one. We need incentives to do our work.” [China]*

Additionally, the presence of the wrong kind of policy was also a barrier. Practitioners in Australia and Brazil described policies in their workplaces that limit their authority to select the best interventions or to make other changes necessary (e.g., enacting a quality improvement system) to incorporate EBCDP as roadblocks for the implementation of EBCDP interventions.

*“I would say one of the most challenging aspects is that you can have evidence coming in at the work level that requires an adaptation that hierarchically needs to go through an approval process, and sometimes the process is so convoluted and slow that it really limits your ability to respond to the context in which you are working.” [Australia]*

*“The issue is the lack of authority to select the best programs. Despite having a specific sector of health surveillance for chronic disease we do not have a lot of autonomy.” [Brazil]*

#### Lack of organizational resources

Practitioners in Brazil, China, and the United States cited an inadequate number of staff to implement EBCDP interventions, and at times too few staff was coupled with the responsibility of serving too large of a jurisdiction. Unsurprisingly, a lack of funding to hire additional staff was a mentioned cause for too few staff. One practitioner from China also cited the low pay compensation of public health practitioners as one reason the community hospitals were under-staffed.

*“Chronic disease management and prevention requires a lot of work, especially for China that has a large population. First, the personnel and money that we can invest in this work are limited. Chronic disease management mainly requires a change of lifestyle and health behavior; this is going to take a long time. The follow-up work, health education, things like these also require a lot of staff-time investment.” [China]*

*“I think some of it is tied back to my earlier comment, which is the resources because if we had more staff, I would have more time to be able to better integrate it.” [United States]*

Despite the many channels through which practitioners in all four countries learn about EBCDP interventions (discussed above) a few practitioners in Australia and China cited lack of access to evidence as a barrier to implementing evidence-based interventions. Several practitioners also pointed to the lack of evidence relevant to rural communities in the United States as barriers. This lack of access or lack of relevant evidence could contribute to under-use of evidence-based repositories in many programmatic areas.

*“…we are, again, a very small, rural community. Some of our towns have less than 1,000 people living in them, so you know it does make it a little bit difficult when looking at the various interventions--is it going to fit the needs of our population?” [United States]*

### Facilitators to implementing evidence-based interventions

Among practitioners in all four countries, two facilitators emerged: education/experience and partnerships. Practitioners in the United States also cited funding agencies that require EBCDP interventions as effective facilitators to the implementation of EBCDP interventions.

#### Education/experience

Practitioners in Australia, Brazil, and the United States included having an education/degree (usually a master of public health) and experience as important facilitators.

*“At a personal level for myself, I've been very fortunate to be supportive and just finished a master’s in public health. I had quite a strong grounding in the social epidemiology.” [Australia]*

*“If I don't know, I seek the information through articles, websites, Google and I seek knowledge from the health department.” [Brazil]*

*“Yea, I would say confidence, experience, I also have a great support system with creating healthy communities.” [United States]*

#### Partnerships

Reminiscent of the importance of networks and peers described earlier, practitioners named partnerships with key organizations and individuals as helpful to the implementation process. These key organizations included: universities, medical schools, coalitions, government agencies and other organizations with political influence on local, state, and federal policies. Partnerships allow practitioners to access databases of EBCDP interventions, expertise on various topics, funding, and political support. They also gained advocates for their work, the ability to influence the curriculum/training delivered to healthcare providers, and accountability for delivering EBCDP interventions.

*“We have our Creating Healthy Communities coalition and Safer Schools is a part of that coalition. There were not any mandates for Creating Healthy Communities or Safe Schools, but everyone who was involved in both of those had a lot of experience and knowledge.” [United States]*

*“Yes, and my whole job is about partnerships and networks and how we support and it is about working about policy practice and research and that's part of what my role was trying to do I guess. It's working with the policymakers that count, so it's working with researchers at different universities and it's working with practitioners…” [Australia]*

*“The federal government finances these processes and the State Department send financing for Municipality Department that implement the programs. Another option is creating partnerships between public and private sectors, but responsibility lies with the Municipality Department.” [Brazil]*

Practitioners received support from colleagues, staff (this includes having an ample number of staff and staff who are supportive in nature), and administrators/leaders within their organizations, as well as support from elected officials. Elected officials were likely mentioned because of their influence on the funding streams and policy environment, especially as they relate to the prioritization of EBCDP. Elected officials can be both barriers and facilitators of EBCDP depending on their values.

*“It [a particular program] used partnerships as the basis for the intervention and that allowed us to achieve more collaborative and coordinated actions. I think there is a strong evidence-base for that.” [Australia]*

*“The support comes from all three levels of government (municipal, state, and federal). One helps with financing (federal), one with structure (state), and the other with work team (municipal).” [Brazil]*

*“Support from administrators/managers within our health department and partnerships or coalitions with other organizations are the primary pieces.” [China]*

*“Partnerships and coalitions, so we have both some support and some barriers from elected officials. And having a partnership and doing this in a way that is in a coalition and having partners that aren’t just the public health department make it much easier to put forward more progressive campaigns. And by campaigns, whether it’s an actual awareness campaign or a campaign to move a policy forward or the movement in general, the larger campaign to reduce sugary drink consumption. It’s absolutely, absolutely, 100% vital to do this in a partnership with people from the community who are impacted as well as other professional organizations.” [United States]*

## Discussion

Several studies have quantified the profound burden of chronic disease in Australia, Brazil, China, and the United States and called for a global public health response [25, 26, 56–58]. Increasing the D&I of EBCDP interventions is an effective method for addressing this public health problem, making efficient use of limited resources, and minimizing harm [59–61]. However, without a shared approach and understanding of global barriers and influences, EBCDP will continue to face challenges. These challenges impede the uptake of effective preventive efforts, contributing to the global rise in chronic disease prevalence and premature death. This study identifies shared barriers to EBCDP across countries that ultimately limit the usefulness and impact of EBCDP on global health. This study also identifies common facilitators that can be used to enhance public health practice across all four countries and improve health equity by further lifting the practice of EBCDP in middle-income countries to the levels of higher-income countries. This study is novel in its aim to qualitatively explore contextual similarities and differences related to the D&I of EBCDP across Australia, Brazil, China, and the United States by interviewing practitioners working in chronic disease prevention in each country. Several key similarities and differences were identified related to how EBCDP interventions are accessed, the use of repositories of evidence-based interventions, and personal and organizational-level barriers and facilitators that influence the D&I of EBCDP. When interpreting the findings, it is important to keep in mind the contextual conditions for EBCDP across the four countries. For example, many of the concepts of EBCDP are newer in Brazil and China making resources and commitment to the concepts covered in our study less available. In addition, the healthcare systems vary widely on a spectrum from highly centralized (China) to highly decentralized (United States), a difference that likely largely influences the barriers to EBCDP experienced by chronic disease prevention practitioners. These differences are likely to influence how clinical practitioners interface with the public health system. Despite these systems-level differences, practitioners across the countries share several barriers and facilitators to EBCDP implementation.

### Access to and use of EBCDP interventions

While practitioners across the four countries indicated that they used several methods for finding EBCDP information, academic journals were the most commonly cited channel through which they access EBCDP information. This is consistent with the literature from Australia and the United States, and a novel finding describing practitioners in Brazil and China [19, 22, 23, 62]. Despite practitioners most commonly accessing EBCDP information through academic journals, peers were cited as the most *useful* channel for accessing EBCDP information. This finding is aligned with Word-of-Mouth Marketing Theory, which posits that peer-to-peer communications and recommendations have powerful influence on decision-making whether its deciding to buy a certain product or implement a certain intervention [63, 64]. A U.S.-based study of public health nurses cited “colleagues as the most efficient and trusted source of information.” [62] Similarly an Australian study of local government public health officers cited managers and personal experiences as the *most useful* people/groups in public health decision-making [65]. Additionally, a social network analysis found that public health practitioners look to their peers within and across divisions to identify relevant evidence [66]. This finding highlights the need for continued support for public health practitioners to attend conferences and other networking meetings that present opportunities to interact as a means for disseminating EBCDP interventions. Through these peer interactions, practitioners in this study describe that they learn about interventions with which others have had success and/or the newest EBCDP interventions that may have not yet been published in academic journals. Conferences and networking meetings may be a helpful, universal strategy for lessening the wide gap in time between research publication and the implementation of findings into practice [19, 62]. Additionally, an online network for sharing ideas could serve as a promising solution for sharing ideas across countries or large geographic areas even within countries, especially due to high costs associated with conference attendance. In contrast, knowledge translation literature out of Australia has found that online information sharing forums have been consistently under-utilized [67]. Promotion of the online forum by organizational leadership may be key to increasing frequency of use [67]. This study presents an incongruence between which channels practitioners use most often for accessing EBCDP interventions and the channels they find most useful. Future research could explore reasons for this difference and how to potentially address it.

The authors had assumed from the greater number of EBCDP publications deriving from Australia and the United States, compared with the few from Brazil and China, that practitioners from Australia and the United States would report more widespread use of repositories of EBCDP interventions (e.g., Guide to Community Preventive Services) (United States), Health-Evidence.org (Australia), Cochrane Collaboration (United States, Australia)) in their workplaces than those from Brazil and China [68, 69]. However, there was surprisingly little variation in the responses across practitioners from the four countries in their low perceived use of the repositories within organizations in which chronic disease prevention work is carried out. Several studies suggest lack of access to research is a significant barrier to evidence-based practice [62, 69, 70]. However, while many participants in this study reported having ample access to key EBCDP resources, this study suggests that access is not enough, evidenced by low use of repositories. Knowing about and navigating the array of reliable, credible web-based public health information resources can be daunting, especially amongst those with little formal training in public health [70–72]. Repositories for EBCDP interventions were created in order to lessen practitioners’ difficulties related to accessing academic journals and siphoning through an overwhelming amount of research, yet these barriers remain and repositories are under-utilized, similar to online networks [67]. The organizational-level barriers (e.g., unsupportive workplace culture, leadership, or policies, and lack of resources) identified in this study could also contribute to the limited use of repositories of EBCDP interventions in workplaces. Like online forums for networking with peers, studies have shown that active organizational efforts to facilitate use of repositories is necessary [73, 74]. The low use of repositories of EBCDP interventions could also reflect the earlier finding that practitioners believe in-person interactions with peers to be more useful than online channels for learning about EBCDP interventions. When frequent peer interactions are not possible, particularly in rural areas and when funds are limited, use of EBCDP repositories may be increased with additional training, especially aimed at organizational leaders. These trainings could focus on the importance of using evidence in decision-making, where to find this information presented in user-friendly ways, and the phases of one’s work at which it would be most helpful to consult such resources.

### Patterns in barriers to the D&I of EBCDP

Limited time and heavy workloads are personal barriers consistent with the literature on barriers published in Australia and the United States [53, 75–77]. Limited time was also identified as a personal barrier among practitioners in Brazil and China, lending credence to this barrier as a more global hindrance to the D&I of EBCDP rather than one specific to higher-income countries. This lack of time could apply to a variety of steps within the implementation process for different practitioners, but limited time to keep up with the latest evidence was mentioned on more than one occasion by practitioners in China. Studies have shown that practitioners in the United States find government reports (e.g., Institute of Medicine Reports) or other summaries (e.g., the Center for Training and Research Translation, Cochrane Collaboration) of EBCDP interventions to be helpful tools for staying abreast of the dynamic field given ever-present time constraints. Practitioners in Brazil and China indicate a potential opportunity to build organizational capacity through staff training on the evidence-based decision-making process and developing EBCDP interventions relevant to the populations they serve. Numerous studies in the United States, Australia and other countries have found that providing training to public health practitioners on the evidence-based decision-making process is an effective method of increasing practitioners’ knowledge, skills, and confidence to use the evidence-based processes [50, 78–83]. Based on the literature related to barriers and capacity building to overcome these challenges, it important to note that individuals shape organizations and organizations support the development of individuals and their skills [84].

While the barriers to EBCDP implementation can be distinctly categorized as personal or organizational barriers, there are clear linkages across these levels. For example, while time and workloads are considered personal barriers, an inadequate number of staff was a common organizational-level barrier that likely exacerbates these personal barriers. Likewise, inadequate funding to hire new staff members, especially staff members with training in public health, was identified by practitioners in this study and other studies as an underlying reason for the under-staffing and/or the lack of training among staff, seen to be problematic to the implementation process across all four countries. Since the scope of this study focused on personal and organizational-level barriers, additional research on the political and sociocultural barriers that influence funding for EBCDP across the four countries is needed. That said, these types of barriers tend to be particularly challenging to change, so it may be more realistic to focus on the organizational integration of strategies that increase the D&I of EBCDP so that practitioners are better supported regardless of the funding environment.

An unsupportive workplace culture was mentioned by practitioners across all four countries as an organizational barrier to the D&I of EBCDP, similarly described in studies from the United States and Australia [20, 23, 51, 77, 79]. However, there were differences by country relating to specific characteristics of this unsupportive culture as well as other organizational barriers. Surprisingly to the authors, there were no clear trends in barriers mentioned by practitioners in middle- versus high-income countries.

There was notable universality of facilitators to the implementation of EBCDP interventions across countries. Education or training was named a facilitator of EBCDP intervention implementation among practitioners from Australia, Brazil, and the United States. On the one hand, Brazilian practitioners’ confidence and fewer perceived barriers is surprising since evidence-based practice is seemingly less established in Brazil relative to the United States and Australia based on empirical literature. On the other hand, lack of expertise with developing and implementing EBCDP among practitioners in Brazil could understandably translate to practitioners’ inexperience with having to confront and identify barriers to carrying out EBCDP. Another potential explanation for why practitioners in Brazil reported fewer personal barriers to implementing EBCDP interventions is that there were more practitioners interviewed in Brazil who had a PhD than the practitioners from other countries. The additional education in these cases may lessen the personal and organizational barriers that others experience, due to extra training or a workplace position that holds more autonomy and authority to make decisions, a barrier among other practitioners from Brazil, as well as Australia and China. Similar to this finding, a quantitative U.S.-study found a significant inverse relationship between education level of public health practitioners and their likelihood of reporting inadequate skills in developing evidence-based interventions [20]. Assessing the extent to which perceptions differ within and between job positions and country is a recommended next research step.

### Partnerships as key facilitators

Partnerships, whether they were individuals or organizations, stood out as the most consistent, powerful facilitator of EBCDP intervention implementation across all four countries. This finding extends the previously discussed usefulness of peer interactions for the successful dissemination of EBCDP interventions to their usefulness for the successful implementation of EBCDP interventions as well. The findings from this study encourage continued and/or increased support for opportunities for public health practitioners to connect with not only one another, but those working in universities, government, private and public sector organizations as an effective means for supporting the D&I of EBCDP interventions across countries. Other studies have identified characteristics and practices that may enhance the effectiveness of partnerships including but not limited to having a vision/mission for the partnership; including partnerships from a wide range of sectors; engaging and empowering community members; systematic action planning, process evaluation, and tracking of outcomes; sharing financial and human resource investments; providing technical assistance and support [46, 85–87].

The importance of interactions and communication with others (a barrier to implementation mentioned by practitioners in China and the United States) within and across organizations to the effective D&I of EBCDP is the chief underlying message shared by practitioners working in chronic disease prevention across the four countries represented in this study. This message is consistent with a systematic review of studies that examined factors that influence the use of evidence by policy makers in middle- and high-income countries [70]. However, the systematic review included only one study from Brazil and none from China. This study’s findings also draw attention to the potential value in analyzing social networks of practitioners within and across countries working in chronic disease prevention in order to improve the D&I of EBCDP [66, 88]. Partnerships and communication among peers are examples of common facilitators to EBCDP that can be fostered to enhance public health practice across all four countries and more effectively prevent chronic diseases. Specifically, the interface between public health and clinical practitioners is likely essential for progress considering primary and secondary levels of chronic disease prevention and the typical settings each tend to be delivered (i.e., community vs. clinical) [89].

### Strengths and limitations

This is the first qualitative exploration of several key constructs of EBCDP D&I across middle- and high-income countries. This study provides insights to multi-level barriers and facilitators to EBCDP intervention implementation by practitioners across four countries. This work can inform larger, population-level research related to the variable and shared contextual factors that influence the uptake of EBCDP interventions with an increasingly global perspective.

While the sample size is adequate for the scope of this study, expanding the scope of the study to systemic contextual factors that influence EBCDP would increase the complexity and likelihood of a larger necessary sample. Due to vast structural differences in the public health delivery systems across the four countries, there was no one workplace or position held across the countries that was exactly equivalent in all countries. The selection of interviewees was susceptible to selection bias; there could be differences between those who agreed to be interviewed and those who did not. The interview data were collected over the telephone in Australia, Brazil, and the United States, but face-to-face in China, due to differences in cultural acceptability and appropriateness. Different methods of data collection present risks of methods-specific biases, for example, telephone interviews do not allow for the visual appraisal of the practitioner’s environment nor the practitioner’s non-verbal communication (e.g., body language) that might otherwise prompt an interviewer to ask probing follow-up questions [90]. Face-to-face interviews have strengths that telephone interviews do not, but face-to-face interviews also pose a heightened risk for social desirability bias compared with telephone interviews, which provide an additional layer of anonymity. Furthermore, social desirability bias is also culturally influenced (i.e., more salient in some cultures than others), which is an additional limitation of this cross-cultural study [91].

## Conclusions

This qualitative study, to our knowledge is the first of its kind, begins to unravel the dynamics and complex interaction of the personal, organizational, and inter-organizational factors that influence implementation of EBCDP across four countries. In general, practitioners from all countries tended to agree that implementation of EBCDP is lacking, as evidenced by the limited use of EBCDP repositories and the many barriers cited to EBCDP implementation. This finding aligns with similar conclusions drawn elsewhere in the literature. This is important because public health resources are almost universally limited, the stakes involved in their misuse are high in terms of human cost/harm, and such misuse occurs when funds are allocated to non-evidence-based programs [3, 92]. The rich findings highlight many contextual similarities and differences with EBCDP that can inform both cross-country and country-specific research and practice to address barriers to EBCDP D&I. For example, key barriers (e.g., lack of expertise for implementing EBCDP) need to be addressed differently in Brazil and China than in Australia and the United States. Interview responses, especially from public health practitioners in Brazil and China where EBCDP is a newer concept, provide insights for designing quantitative measurement instruments for future population-level, cross-country research and evaluation.

## Abbreviations

D&I: Dissemination and implementation
EBCDP: Evidence-based chronic disease prevention
